# Tobacco

**Published:** 1880-02

**Authors:** T. B. Spalding

**Affiliations:** Troy Ill.


### TOBACCO.

BY T. B. SPALDING, M. D. OF TROY, ILL.*

*An Address before the Madison County Society.*

In a recent essay before this society, I considered the action of alcohol within the human system, and on this occasion I am pleased to respond to your courteous invitation with observations on the action of tobacco. These agents might be profitably presented as almost identical in action, and shown to be largely accessory to each other's sins, but the *temperance* is waived for the *physiological* phase of the argument.

Of tobacco's origin, its introduction, its composition, its cost, the extent of its consumption, and the processes of its preparation, I purposely pass, to deal more directly with it in its physiological relations to the functions and forces of human life.

Eminent authority in every country and in every department of science, concur in classing tobacco among the narcotic poisons, than which none are more deadly; indeed, like Aaron's rod, it has secure within itself, the most magical and worst of all its rivals. Nicotia, sulphureted hydrogen, hydrocyanic acid! What a den of deadliest poisons, all having their *habitat* in this colossal curse, termed tobacco.

A poison is declared to be "anything whose natural action is capable of producing a morbid, noxious and dangerous effect upon the organization of anything endowed with life." Thus we perceive the definition is the perfect picture of tobacco's action. Acquainted with this agent for over two hundred years, medical science, speaking with the tongue of every science, declares tobacco wholly innutritious, and further still, declares it nauseous; not only that, but noxious; and further yet, a repository of deadliest poisons. From this dictum, there is no appeal; in its truth, medical men are forced by their culture, to concur. But even then they dandle with Delilah till shorn of strength, and science must still be summoned and held aloft for the healing of the nation. If tobacco is a poison, it ought to act as such, and it may be safely affirmed, it *has no other action*!—no other use in medicine, than to depress vitality. Thus it nauseates, it paralyzes the nerve centers, producing relaxation of the muscular system, and produces such dreadful prostration that medical literature is full of warning, and abounds with reported cases of fatal poisoning by this agent. When medical science was in her cradle, and chloroform in the embrace of chaos, ere anæsthetics had come, as the olive leaf dove, to the ark of Æsculapius, surgeons soothed their suffering patients with powerful potations of tobacco, and thus they utterly prostrated the vital powers, relaxed the muscular system, and then proceeded to reduce luxations! How direful must have been a patient's difficulty, if half so dreadful and distressing as the remedy.

It may be affirmed and demonstrated of tobacco, what is strikingly exceptional, namely, that it alone, of all the vegetable kingdom possesses two active principles—the one an alkaloid, and the other an oil, and both the deadliest of poisons.

It has been urged in support of fashionable poisons, that because multitudes use them, therefore they can't be especially dangerous; but professional science and experience teach that there isn't an agent in the entire armory of toxicology, but the human system, by continued use, may at length be brought to tolerate it.

One-fifth grain of strychnia, or one grain of morphia, will destroy life, yet, by constant and long continued use, the blunted susceptibilities of the nerve centers may be made so to tolerate these and like poisons that eventually enough may be taken to destroy fifty men. It is demonstrated in the observation of every one that the use of noxious agents, especially tobacco, begets a morbid appetite which demands that continually more of it *may* and *must* be employed to produce the same impression.

Such we know are facts respecting what is noxious, but is not the case with what is nutritious. Medical science is not satisfied with *statements*, but sounds the depths in search of a *philosophy* for asserted facts, and she declares, in this regard, that nutritious agents create and renew nerve cells and structures, and endow them with the finest physiological sensibilities, while noxious agents disturb the conditions essential to their renewal, and so benumb and paralyze their normal sensibilities, and produce, inevitably, the pathological and characteristic condition of requiring continually more of the disturbing poison, to produce the same impression. With these truths, we enter the most fascinating field in nature, to consider the conduct of this agent in the laboratory of life. Nowhere has Deity evinced such evidences of an intelligent, divine supernatural as here presented in the adaptation of means to ends—in the perfect play of affinities and forces ever operative in the construction and destruction, the waste and renewal of this physical citadel that enshrines an immortal soul. The whole sublime, but sensitive train of transition involved in the conversion of solid food, first into fluidity, and under the auspices of vital force, transformed upward through intricate gradations till it attains the climax of its course in other solid forms, either of flesh or bone or brain, and then the oxidation of these, and the evolution of heat and force, is the perfect process of what we term digestion. The brain is the depot of life's dynamics! It is the sun of the physiological system, which, with its accessory centers and nerve cords, receive and transmit to the system, a force that propels the mightiest and minutest processes of physical life.

But the ability of these organs—as instruments of the mind—thus to receive and transmit this vital force, depends essentially on their structural health and perfection. Paralyze or impair the perfection or structural integrity of the brain, disturb the subtile harmony of those changes of waste and renewal ever operative and essential to its structural perfection, and at once its power is impaired to forcibly and healthily perform its functions; and this adverse influence is precisely the action of tobacco as a depressing poison. The proposition is plain, the truth is self-evident and irresistible, that, with the nerve centers thus benumbed and blighted, and the vital force impaired, then every digestive processs dependent on the harmonious action of vital force, is weakened and discordant, and the physical and mental man is deranged to the extent that the physical machinery is injured.

The noxious influence of tobacco is more actively operative upon one class of persons than upon others. I may, therefore, for convenience, divide the victims of tobacco into two classes, assigning to the first class, all those who do manual labor. These suffer least from fashionable poisons, because the deadening influence of noxious agents upon the nervous system is largely counteracted by physical toil, which strengthens the entire system and conduces to health ; and thus it is that active poisons are thought to "kill slowly," and laboring people live long, apparantly uninjured, and practice poisonous indulgences. In all this great and glorious class of humanity, however, may be found the fruits of tobacco's use, in the form of cancer on the lips and tongue, dyspepsia, constipation, and hemorrhoids. But let us consider the other class, wherein are included ladies and gentlemen of wealth, of fashion and of leisure, those who live idle as well as those devoted to literary pursuits and purely sedentary occupations. Physicians, ministers and lawyers are of this class, and in all these we find paralysis very prevalent, and that diversified and interminable train of nervous derangements whose name is legion. With constitutions enfeebled by physical inactivity and sensibilities heightened by social and literary culture, consider, for a moment, the effect upon these highly nervous natures. To all of this priceless portion of humanity the use of tobacco is unmixed evil and rapidly ruinous.

Again, it is affirmed by eminent authority that tobacco is the prolific, if not indeed, the only source of delirium tremens.

First, the ancients were entirely unacquainted with these terrible terrors of the inebriate, and the records beyond the discovery of tobacco (1560) reveal no case of *mania a potu.*

Second the normal action of tobacco, is the production of tremens, and the most frightful forms of delirium tremens are daily produced by the use of tobacco alone.

Third, it is rarely possible to find an inebriate who does not use also tobacco, and careful inquiry will confirm the statement that, with 90 per cent of such cases, the tobaccó habit was first formed. Its influence deranged the nerve centers, an initial tremens was entailed upon the nervous system, which suggested to the morbid taste of the sufferer, the soothing, sedative action of alcohol, and thus the allied agents forge for each other and fasten more firmly, the chains of the most servile slavery.

I have employed professional science to loosen the pillars of tobacco's position, and with authority and with argument, have carefully criticised its action and influence on the functions and forces of organic life. Earnestly in this direction I invoke the sober judgment of scientific medicine, and when you shall have ordered tobacco to abdicate, then only will it fall from popular use and favor, and with that will end the ruin it has wrought.

In view of these truths, scientific and self-evident, in the name of science that classifies all knowledge, in the name of science that seeks the essential nature of things, in the name of science that truthfully interprets the teachings of nature, issue the edict of your eminent authority, and drive from popular use and favor this poisonous plague, and when this is secured, a heavenly halo of light, an ineffable effulgence will open up over the poisonous wastes of the world, a broad, and bright and beautiful pathway of crimson and of gold, wherein garlanded angels will gladly gather, proclaiming "peace on earth and good will toward men," and from highest heaven all over the earth shall you cause to be heralded God's emancipation proclamation to a world that is wasting its highest and holiest possibilities in the ruinous depressing practice of popularized poisons.

